# Metachronous Subareolar Pleomorphic Adenoma and Contralateral Invasive Ductal Carcinoma of the Breast: A Case Report

**DOI:** 10.70352/scrj.cr.26-0491

**Published:** 2026-11-12

**Authors:** Rieko Tsuru, Yoshika Nagata, Izumi Kinoshita, Toshihiro Saeki, Takahisa Fujikawa

**Affiliations:** 1 Department of Surgery, Kokura Memorial Hospital, Kitakyushu, Fukuoka, Japan; 2 Department of Pathology, Kokura Memorial Hospital, Kitakyushu, Fukuoka, Japan

**Keywords:** pleomorphic adenoma, breast cancer, ductal carcinoma *in situ*, bilateral breast tumor, surgical resection

## Abstract

**INTRODUCTION:**

Pleomorphic adenoma (PA) of the breast is an extremely rare benign tumor. A preoperative diagnosis is challenging as its clinical and radiological features often mimic those of malignancy. We report a unique case of metachronous bilateral breast tumors consisting of PA in one breast and invasive ductal carcinoma (IDC) in the contralateral breast.

**CASE PRESENTATION:**

A 70-year-old woman presented with nipple discharge 18 years earlier. Imaging findings suggested a benign papillary lesion near the left nipple, and the lesion was managed with clinical observation. Ten years earlier, the tumor had enlarged, prompting a needle biopsy that yielded a diagnosis of a benign papillary lesion. At the current evaluation, a new lesion with calcifications was detected in the right breast, and further investigation confirmed ductal carcinoma. This diagnosis prompted a re-evaluation of the left breast tumor. A repeat needle biopsy could not definitively distinguish between a benign lesion and a malignant lesion. Therefore, a surgical biopsy of the left breast tumor was performed. Histopathological examination revealed epithelial and myoepithelial proliferation with duct formation and gradual transition into a chondromyxoid stroma, consistent with PA of the breast.

**CONCLUSIONS:**

Primary PA of the breast is rare and can be radiologically misdiagnosed as a malignant lesion. In the present case, the PA and IDC were considered biologically unrelated lesions that arose independently and coincidentally in the contralateral breasts. Nevertheless, when evaluating bilateral breast lesions, particularly in the presence of a malignancy in the contralateral breast, clinicians must proceed with diagnostic caution to avoid overtreatment.

## Abbreviations

ACR
: American College of Radiology
BI-RADS
: Breast Imaging Reporting and Data System
CNB
: core needle biopsy
HER2
: human epidermal growth factor receptor 2
IDC
: invasive ductal carcinoma
IHC
: immunohistochemical
MeSH
: Medical Subject Headings
MG
: mammography
PA
: pleomorphic adenoma
SMA
: smooth muscle actin
WI
: weighted images

## INTRODUCTION

PA is a benign mixed tumor that most commonly arises in the salivary glands; however, its occurrence in the breast is extremely rare.^1)^ The existence of such tumors in the breast can be attributed to a shared embryonic origin between the salivary and mammary glands.^2,3)^ Breast PA typically develops in the subareolar region and may mimic carcinoma, often complicating the preoperative diagnosis.

We report a rare case of breast PA that presented a diagnostic challenge in the presence of contralateral IDC.

## CASE PRESENTATION

The patient was a woman in her 70s with a medical history of an internal carotid artery aneurysm and a hiatal hernia. There was no remarkable family history. She initially presented with a bloody nipple discharge from the left breast 18 years earlier. At that time, imaging findings suggested a benign papillary lesion, and the lesion was managed with clinical observation. Ten years ago, 8 years after the initial consultation, the lesion had increased in size to 1.0 cm in maximum diameter, and a CNB revealed a benign papillary lesion. The patient therefore remained under follow-up, and no findings suggestive of malignancy were observed for the next 8 years.

MG revealed newly developed polymorphic linear calcifications distributed in a segmental pattern in the upper outer quadrant of the contralateral right breast, classified as BI-RADS category 4 (**Fig. 1A**). US demonstrated extensive ductal dilatation with internal hyperechoic lesions extending from the nipple to the upper outer quadrant. The contrast-enhanced MRI showed a segmental, non-mass enhancement in the right breast, which is categorized as ACR BI-RADS (5th edition) category 5. A CNB confirmed ductal carcinoma of the right breast. Laboratory findings, including tumor markers, were unremarkable. A total mastectomy was performed for the right breast cancer. The disease was predominantly *in situ*, with a 7 mm solid-type invasive lesion. Hormone receptor analysis by IHC showed that neither the estrogen receptor nor the progesterone receptor was expressed (0%). The HER2 score was 3+, and the Ki-67 labeling index was 15%. These findings are consistent with the HER2-positive surrogate subtype based on IHC. No distant metastases were detected, and the clinical stage was classified as stage I.

**Fig. 1:**
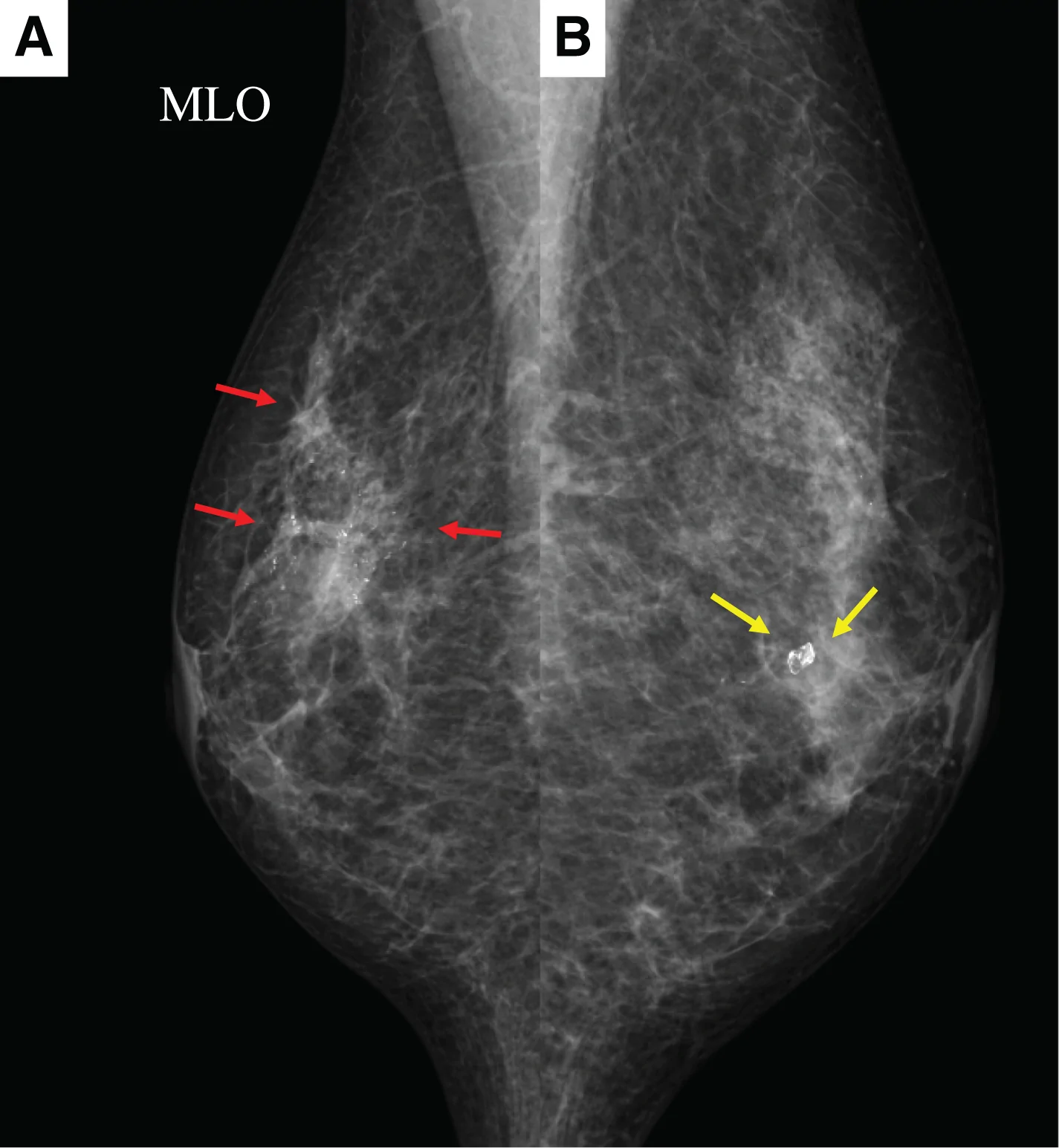
MG image findings. (**A**) MG revealed newly developed polymorphic linear calcifications distributed in a segmental pattern (red arrows), accompanied by architectural distortion in the upper outer quadrant of the contralateral right breast. (**B**) MG showed a focal asymmetry in the middle portion of the left breast (yellow arrows), associated with coarse calcifications. MG, mammography; MLO, mediolateral oblique

Meanwhile, US of the left breast revealed a lobulated, hypoechoic mass with a relatively well-defined margin. The tumor measured 1.2 cm in maximum diameter and showed heterogeneous internal echogenicity with cystic components (**Fig. 2A** and **2B**). MG demonstrated focal asymmetry in the middle portion of the left breast with coarse calcifications, classified as ACR BI-RADS category 3 (**Fig. 1B**). MRI revealed a dumbbell-shaped tumor with iso-intensity on both T1- and T2-WI, with a partial high-intensity area on T2WI. Contrast-enhanced MRI demonstrated heterogeneous enhancement with internal dark septations. The kinetic analysis showed a fast initial enhancement followed by a plateau pattern, corresponding to ACR BI-RADS category 4 (**Fig. 3A**). Additionally, a region of non-mass enhancement was noted on the cranial aspect of the tumor (**Fig. 3B**). Because imaging studies of the left breast lesion showed equivocal findings and could not entirely rule out malignancy, a CNB was re-performed. Although the initial CNB specimen had demonstrated a benign papillomatous lesion (**Fig. 4A** and **4B**), the subsequent CNB specimen exhibited a highly heterogeneous pathological appearance. A dense area containing variable ductal epithelial components and an abundant, hypocellular myxofibrous and myxoid/chondroid stroma was identified; however, a distinct transitional zone between these 2 components was not apparent (**Fig. 4D**). A medium-magnification view demonstrated a distinct biphasic pattern, characterized by inner epithelial cells forming tubules and outer myoepithelial cells proliferating with pale cytoplasm, without significant cytological atypia (**Fig. 4C**). Consequently, differentiating between a benign lesion and a low-grade malignancy posed a diagnostic challenge at the biopsy stage. Because the potential for malignant transformation or a low-grade malignancy could not be entirely ruled out, an excisional biopsy (local excision) was performed for both a definitive diagnosis and treatment.

**Fig. 2:**
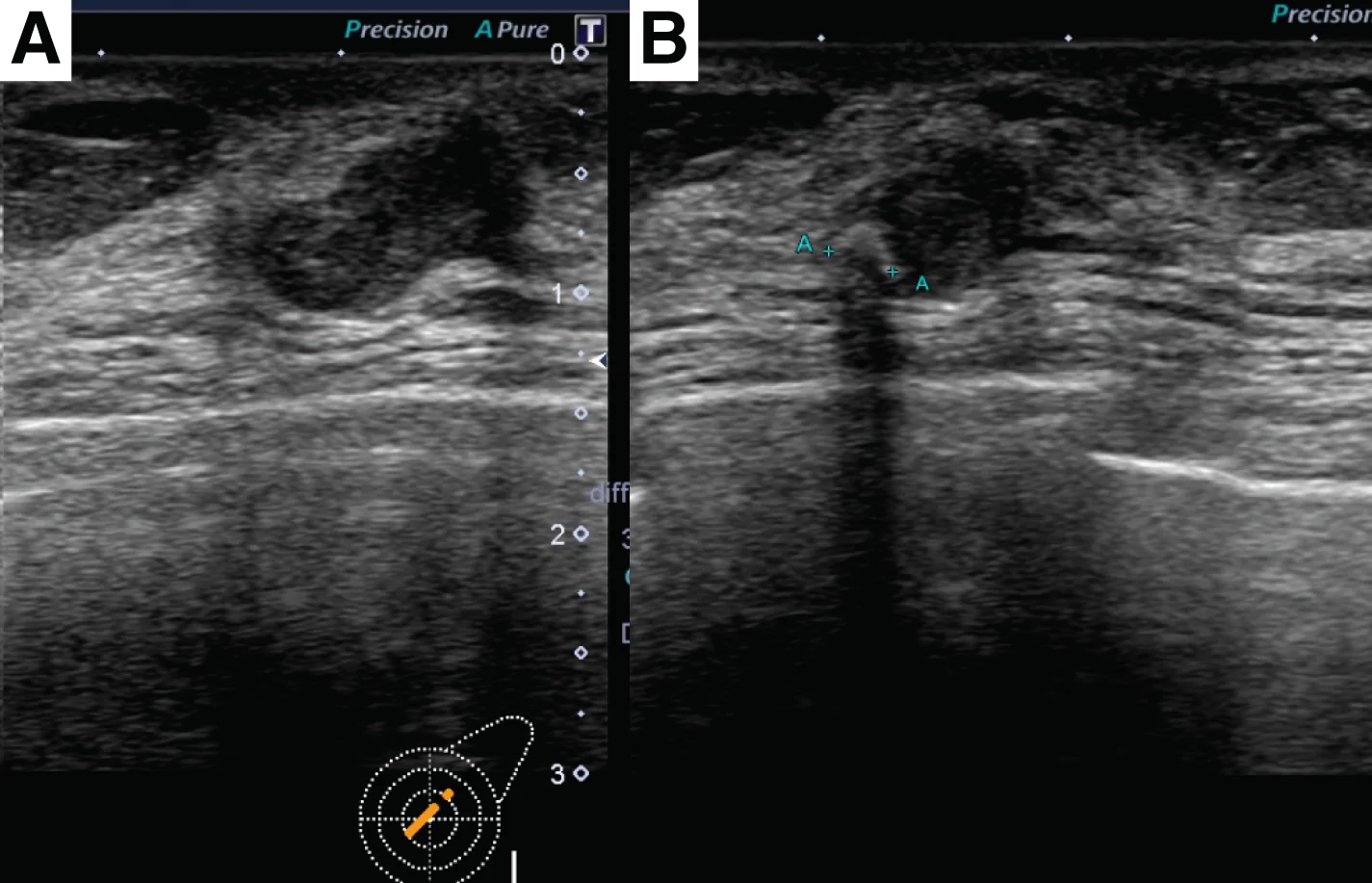
US image findings. (**A**) US of the left breast revealed a lobulated, hypoechoic mass with relatively well-defined margins and cystic components. (**B**) Coarse calcification associated with posterior acoustic shadowing was observed in the lower-left portion of the tumor.

**Fig. 3:**
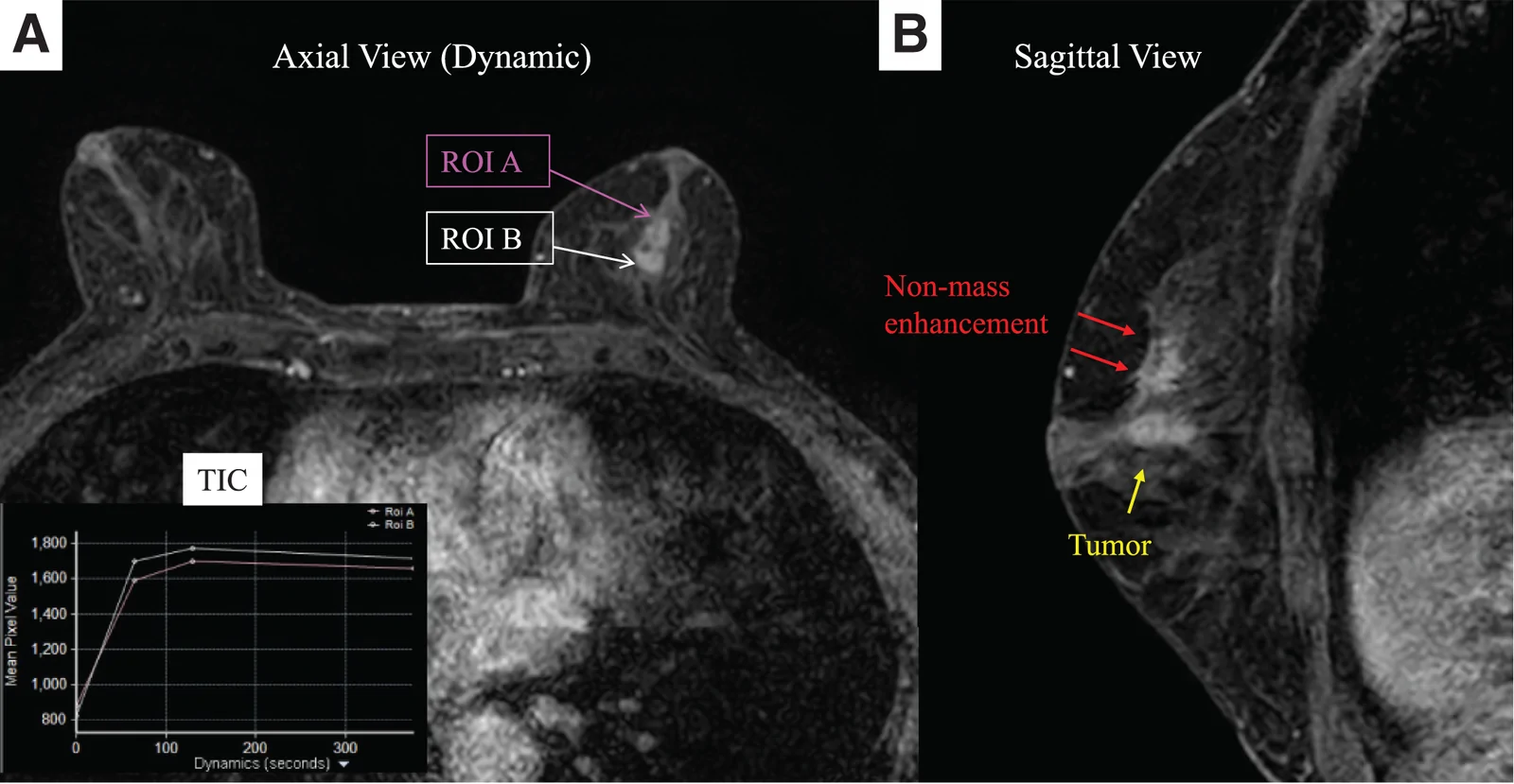
MRI findings of the left breast. (**A**) Dynamic contrast-enhanced MRI demonstrated an early-enhancing lesion with a plateau kinetic pattern on time–intensity curve analysis. ROI A was placed on the nipple-side portion, and ROI B was positioned on the chest wall-side portion of the tumor. (**B**) Contrast-enhanced MRI revealed non-mass enhancement extending toward the cranial aspect of the lesion. ROI, region of interest; TIC, time-intensity curve

**Fig. 4:**
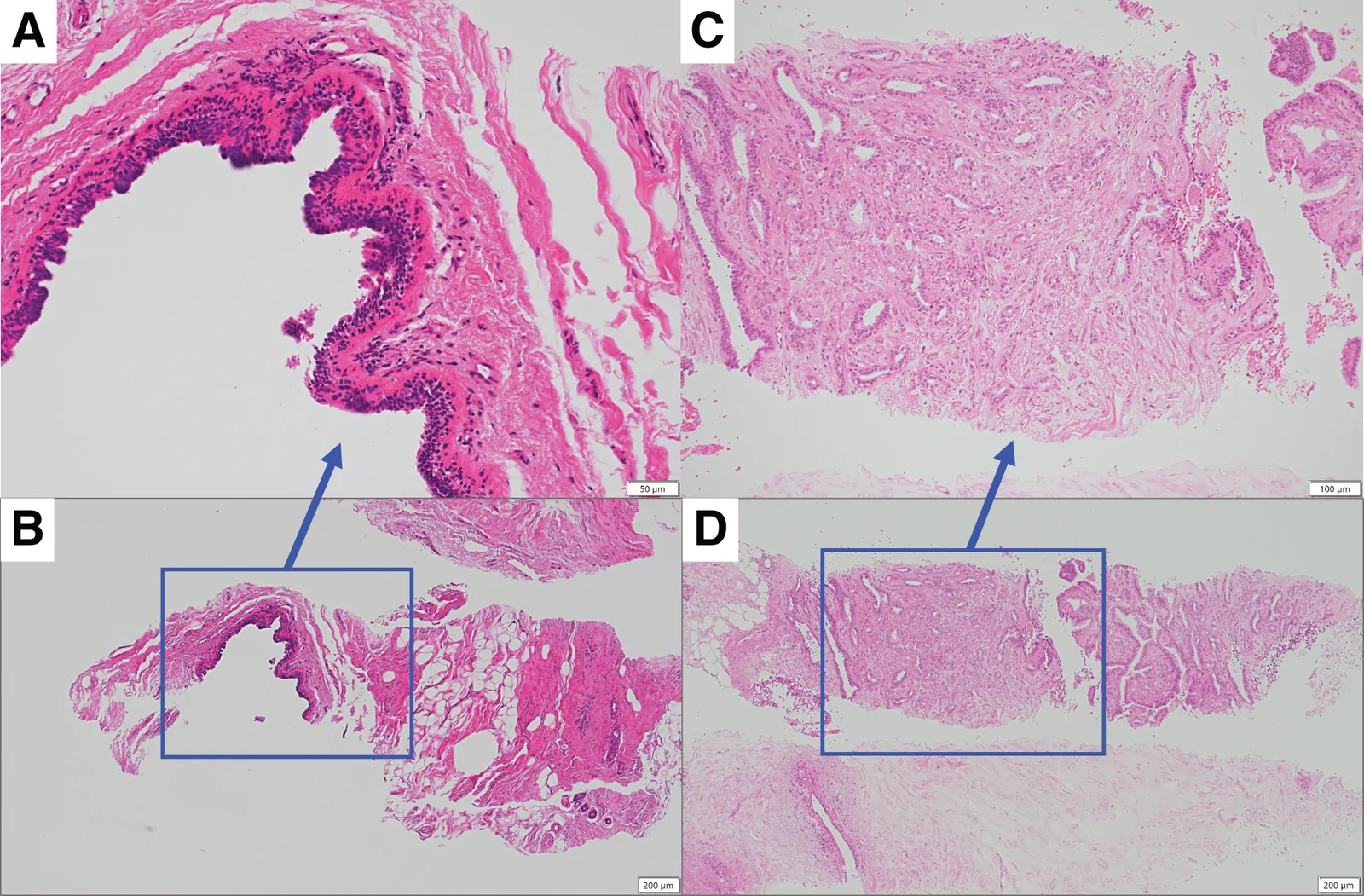
Pathological findings of the CNB. (**A**) Histopathological image of the previous CNB specimen. Higher magnification view of the boxed area in (**B**). H&E staining. Scale bar = 50 μm. (**B**) Low-power view (H&E staining). Scale bar = 200 μm. (**C**) Higher magnification view of the boxed area in (**D**) from the current CNB specimen, showing atypical ductal proliferation (H&E staining). Scale bar = 100 μm. (**D**) Low-power view of the current CNB specimen (H&E staining). Scale bar = 200 μm. CNB, core needle biopsy; H&E, hematoxylin and eosin

Pathological examination revealed a tumor composed of both epithelial and myoepithelial components within a fibrous capsule, accompanied by myxoid stroma and osseous and cartilaginous differentiation (**Fig. 5A** and **5B**). A gradual transition from the myxoid stroma to the cartilaginous component was observed, supporting the diagnosis of PA of the breast (**Fig. 5C** and **5D**).

**Fig. 5:**
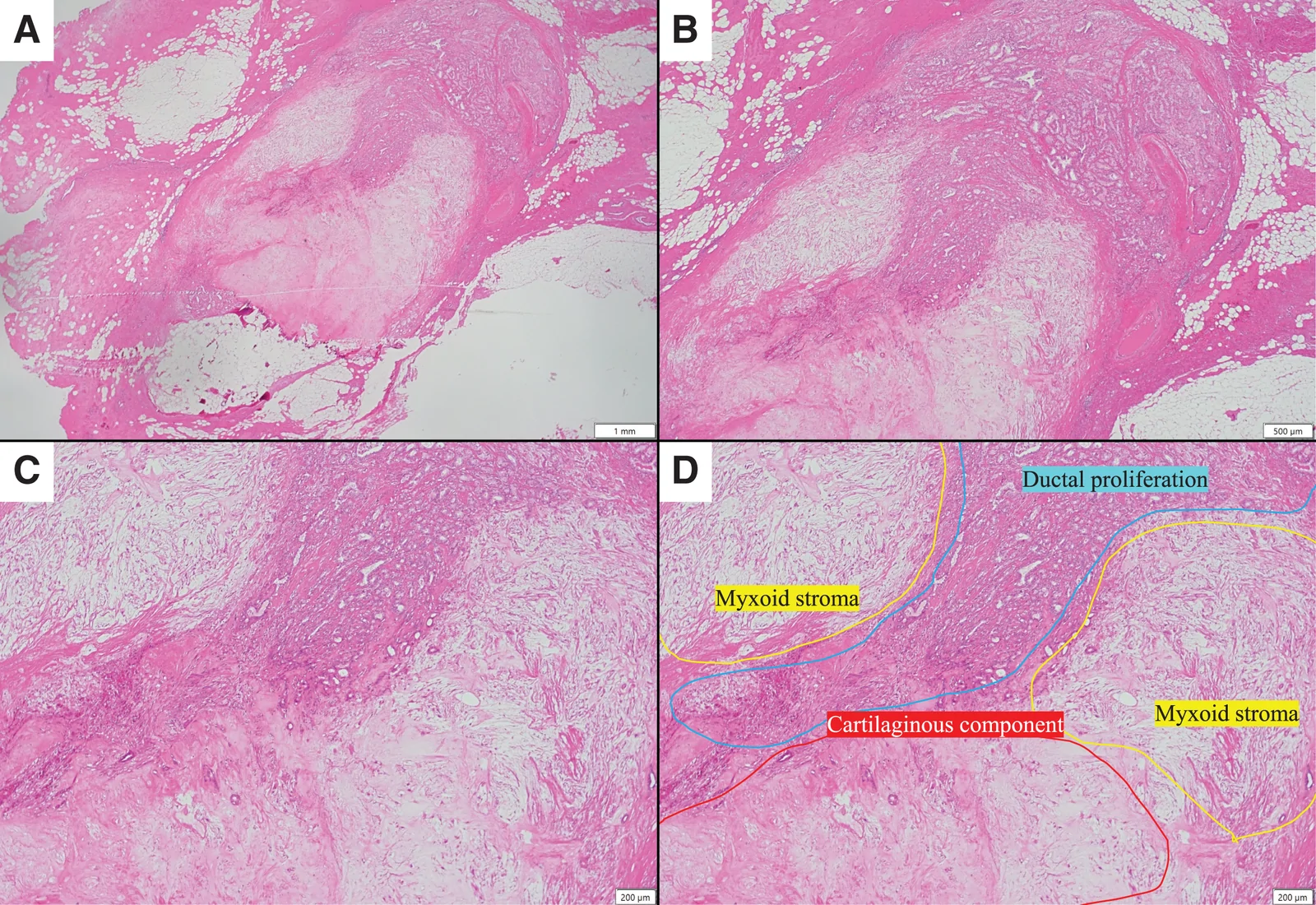
Pathological findings of the resected specimen. (**A**) Low-power view of H&E staining showing a lobulated mass surrounded by a fibrous capsule, with focally ill-defined borders. Scale bar = 1 mm. (**B**) Proliferation of epithelial cells with myxoid and cartilaginous components is observed within the tumor. Scale bar = 500 μm. (**C**) Gradual transitions from epithelial cells to the myxoid stroma and cartilaginous/chondroid regions are observed. Scale bar = 200 μm. (**D**) Annotated image of panel (**C**) showing myxoid stroma (yellow outlines), a cartilaginous component (red outline), and ductal proliferation (blue outline). Scale bar = 200 μm. H&E, hematoxylin and eosin

An abundant eosinophilic chondroid-to-chondromyxoid matrix was observed, containing scattered round-to-oval chondrocyte-like cells within lacunae. These cells had small, relatively uniform nuclei, inconspicuous nucleoli, and scant cytoplasm. There was no significant cellular atypia or tumor necrosis, and it was determined to be cartilage differentiation (**Fig. 6A**). In the ductal structures, myoepithelial cells were identified in the basal layer. In the myxoid stroma, these cells exhibited a spindle-shaped morphology (**Fig. 6B**). Immunohistochemically, the tumor cells were positive for alpha-SMA in both the epithelial and stromal components; however, p63 expression was absent in the myxoid (spindle cell) areas (**Fig. 6C** and **6D**). The absence of significant cytological atypia, together with these findings, supported the exclusion of metaplastic carcinoma. The surgical margins of the excisional biopsy were negative. Postoperative adjuvant therapy for breast cancer was not performed at the patient’s request. The patient has remained free of recurrence during 6 years of follow-up.

**Fig. 6:**
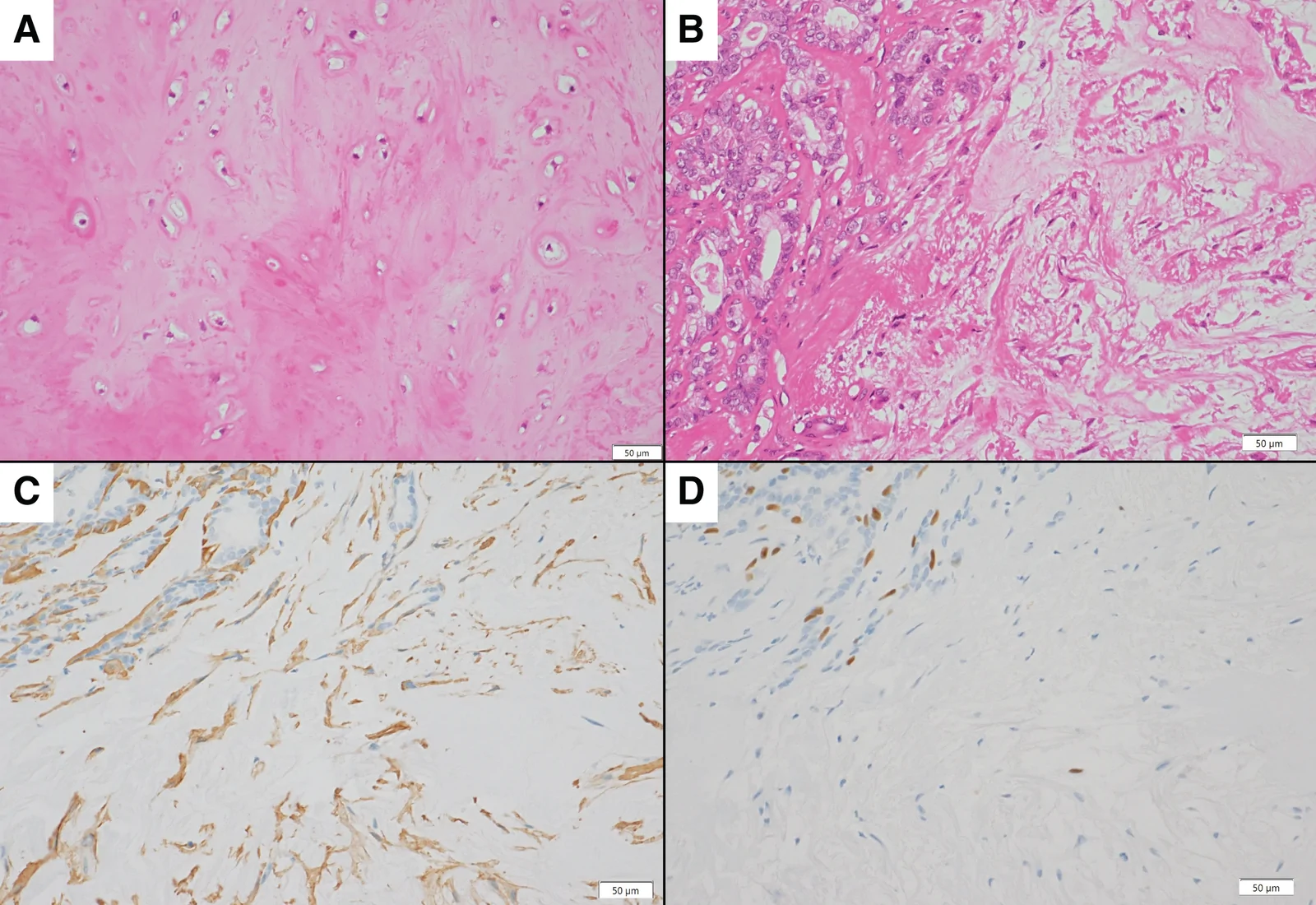
Histopathological and IHC findings of the excised specimen. (**A**) H&E staining showing a chondroid matrix containing scattered chondrocyte-like cells within lacunae, without significant atypia or mitotic activity. Scale bar = 50 μm. (**B**) Myoepithelial cells were identified in the basal layer of the ductal structures and showed a spindle-shaped morphology within the myxoid stroma. Scale bar = 50 μm. (**C**) IHC staining for alpha-SMA. Tumor cells showed positivity for SMA in both the epithelial and stromal components. Scale bar = 50 μm. (**D**) IHC staining for p63. p63 expression was absent in the myxoid spindle cell areas. Scale bar = 50 μm. H&E, hematoxylin and eosin; IHC, immunohistochemical; SMA, smooth muscle actin

## DISCUSSION

PA of the breast is a benign tumor composed of a mixture of ductal epithelial cells, myoepithelial cells, and mesenchymal components, such as myxoid and cartilaginous elements. Histologically, it is similar in morphology to PA that occurs in the salivary glands, and it accounts for a very small proportion of all mammary tumors.^1–3)^ The first case was reported in France in 1906.^4)^ The age range of those affected is wide, from their 30s to their 80s, and it has been reported to be more common in postmenopausal women in their 50s and 60s.^5,6)^

Clinically, PA presents as a painless, well-defined, elastic-hard mass that is usually detected at around 1–3 cm in diameter; however, it can grow slowly and become enormous. Because myoepithelial cells are abundant in the large milk ducts near the nipple, it has been reported that it preferentially occurs in the large milk ducts, such as those in the areola.^6)^

MG reveals a well-defined, circular-to-lobulated mass, sometimes accompanied by calcifications. In this case, MG showed coarse calcifications, which seem to indicate chondrometaplasia when contrasted with pathology. US reveals a well-defined, hypoechoic mass with heterogeneous internal echogenicity. MRI contrast enhancement patterns are inconsistent, ranging from a benign, gradually increasing pattern to early enhancement suggestive of malignancy. Therefore, imaging diagnoses include “fibroadenoma,” “intraductal papilloma,” “phyllodes tumor,” or, if accompanied by calcification or an early enhancement pattern, “breast cancer (e.g., mucinous carcinoma or scirrhous carcinoma).”^7,8)^

Histopathological features often include well-defined boundaries and the presence of a pseudocapsule. A biphasic pattern is observed between the epithelial cells forming the lumen and the myoepithelial cells surrounding it, which results in positivity for epithelial markers (cytokeratin CAM5.2, cytokeratin AE1/AE3, and epithelial membrane antigen) and myoepithelial markers (p63, SMA, S-100 protein, and calponin). The most distinctive feature is that these cells are embedded within a myxocartilage-like matrix.^9)^

Because the amount of tissue obtained from a needle biopsy can be limited, the differential diagnosis varies depending on the puncture site. In cases with extensive myxoid degeneration, the differential diagnosis includes mucinous carcinoma. A papillary growth pattern favors an intraductal papilloma, whereas a phyllodes-like architecture suggests a phyllodes tumor. Furthermore, metaplastic carcinoma and non-neoplastic (reactive) cartilaginous metaplasia should also be considered in the differential diagnosis. Careful confirmation of atypia, mitotic figures, and the presence or absence of invasive growth is necessary. For lesions showing biphasic tumor formation, adenomyoepithelioma and adenoid cystic carcinoma are among the possibilities for differential diagnosis. In this case, a definitive diagnosis could not be established by CNB because the limited tissue sample did not allow identification of the characteristic transition zones showing a gradual transition from the myoepithelial component to the myxoid and cartilaginous components. The final diagnosis was established based on the characteristic biphasic architecture, transition zones, and IHC findings in the excised specimen.

Treatment consists of surgical resection (tumor excision or partial mastectomy), and the prognosis is excellent if the lesion is adequately excised. While caution must be exercised to avoid an unnecessary mastectomy due to preoperative overdiagnosis, incomplete resection carries a risk of local recurrence,^10)^ similar to PA of the salivary glands. Furthermore, it is reported that approximately 6.2% of salivary gland PAs undergo malignant transformation into carcinoma ex PA.^11)^ Given that malignant transformation into carcinoma ex PA has also been reported in extremely rare cases of breast PA,^12)^ surgical excision with clear margins is essential.

In this case, although the breast cancer developed in the patient’s 70s, the PA had been clinically diagnosed as a “benign tumor” in her 50s—the peak age of incidence for PA. The diagnosis of PA was only confirmed upon re-evaluation triggered by the development of contralateral breast cancer. The risk of occurrence and the growth rate of PA may be influenced by changes in the hormonal environment. In elderly patients, even if a CNB identifies a lesion as “benign,” the specific and rare diagnosis of “PA” is often not pursued in further detail. Furthermore, this case involved metachronous PA of the mammary gland and invasive breast cancer. Salivary gland PA and invasive breast cancer that occur simultaneously or metachronously have been reported.^13)^ Although mammary PA may harbor *PLAG1* or *HMGA2* alterations^9)^, these are not established driver mutations for conventional IDC. Furthermore, there is no evidence suggesting that PA increases the risk of contralateral breast cancer. Therefore, the coexistence of these 2 tumors was considered a coincidental event rather than a biologically related event.

The cases of multiple breast tumors, including mammary PA, are summarized in **Table 1**.^14–17)^ A comprehensive literature search was performed in PubMed and Google Scholar from inception to June 30, 2026 (the date of the final search). The search used combinations of MeSH and free-text terms: (“pleomorphic adenoma” OR “benign mixed tumor”) AND (“breast” OR “mammary”) AND (“cancer” OR “carcinoma”). No date restrictions were applied. Eligible cases were selected via full-text review and manual screening of reference lists. In all reported cases, surgical management was individualized based on the location, size, and extent of the lesion rather than being determined solely by the presence of breast cancer. The prognosis was generally favorable. To the best of our knowledge, no previous report has described PA in one breast and metachronous primary IDC in the contralateral breast (**Table 1**). An important clinical lesson is that, even if imaging studies suggest malignancy, one should not assume that the contralateral breast lesion is a metastatic or secondary malignancy. Proper management of multiple breast lesions requires a thorough pathological evaluation and an individualized treatment strategy for each lesion.

**Table 1:** Summary of reported cases of multiple breast tumors associated with PA

Case	Author (references)	Publication year	Age	Metachronous/synchronous	Bilateral/ipsilateral (multicentric/multifocal)	Histology (non-PA lesions)	Tumor size (mm; non-PA lesions)	Surgical procedure (non-PA lesions)	Tumor size (mm; PA)	Surgical procedure (PA)	Treatment (adjuvant therapy)	Outcome (months)
1	Di Bonito M, et al.^14)^	2015	58	Synchronous	Bilateral	IDC, DCIS	10, 10	Radical resection	N/D	Radical resection	N/D	N/D
2	Zhang WT, et al.^15)^	2022	84	Synchronous	Ipsilateral (multifocal)	Malignant AME	22 in total	Partial resection	22 in total	Partial resection	None	18-M RFS
3	Nagasawa S, et al.^16)^	2025	70	Synchronous	Ipsilateral (multifocal)	DCIS	6 × 3 × 2	Partial resection	7 × 6 × 5	Partial resection	None	12-M RFS
4	Guo M, et al.^17)^	2025	96	Synchronous	Ipsilateral (multifocal)	IDC	3	Partial resection	22	Partial resection	None	11-M RFS
5	Our case	2026	70s	Metachronous	Bilateral (multifocal)	IDC (*in situ*: predominant)	7, 4 (*in situ*: 65 × 35)	Total mastectomy	12 × 9	Excisional biopsy	None	72-M RFS

AME, adenomyoepithelioma; DCIS, ductal carcinoma *in situ*; IDC, invasive ductal carcinoma; M, month; N/D, not described; PA, pleomorphic adenoma; RFS, recurrence-free survival

## CONCLUSIONS

Preoperative imaging findings of PA of the breast are often nonspecific, making accurate diagnosis and surgical planning challenging. In the present case, bilateral breast tumors were identified, comprising a malignant lesion in one breast and a PA in the contralateral breast. Given the extreme rarity of this combination, we herein report this case.

## DECLARATIONS

### Availability of data and materials

The datasets of this case report are available from the corresponding author upon reasonable request.
